# Visual detection thresholds in two trophically distinct fishes are compromised in algal compared to sedimentary turbidity

**DOI:** 10.1093/conphys/coy044

**Published:** 2018-08-17

**Authors:** Chelsey L Nieman, Andrew L Oppliger, Caroline C McElwain, Suzanne M Gray

**Affiliations:** School of Environment and Natural Resources, The Ohio State University, 2021 Coffey Rd, Columbus, OH 43210, USA

**Keywords:** emerald shiner (*Notropis atherinoides*), Lake Erie, optomotor response, turbidity, visual sensitivity, walleye (*Sander vitreus*)

## Abstract

Increasing anthropogenic turbidity is among the most prevalent disturbances in freshwater ecosystems, through increases in sedimentary deposition as well as the rise of nutrient-induced algal blooms. Changes to the amount and color of light underwater as a result of elevated turbidity are likely to disrupt the visual ecology of fishes that rely on vision to survive and reproduce; however, our knowledge of the mechanisms underlying visual responses to turbidity is lacking. First, we aimed to determine the visual detection threshold, a measure of visual sensitivity, of two ecologically and economically important Lake Erie fishes, the planktivorous forage fish, emerald shiner (*Notropis atherinoides*), and a primary predator, the piscivorous walleye (*Sander vitreus*), under sedimentary and algal turbidity. Secondly, we aimed to determine if these trophically distinct species are differentially impacted by increased turbidity. We used the innate optomotor response to determine the turbidity levels at which individual fish could no longer detect a difference between a stimulus and the background (i.e. visual detection threshold). Detection thresholds were significantly higher in sedimentary compared to algal turbidity for both emerald shiner (mean_sediment_ ± SE = 79.66 ± 5.51 NTU, mean_algal_ ± SE = 34.41 ± 3.19 NTU) and walleye (mean_sediment_ ± SE = 99.98 ± 5.31 NTU, mean_algal_ ± SE = 40.35 ± 2.44 NTU). Our results suggest that across trophic levels, the visual response of fishes will be compromised under algal compared to sedimentary turbidity. The influence of altered visual environments on the ability of fish to find food and detect predators could potentially be large, leading to population- and community-level changes within the Lake Erie ecosystem.

## Teaser

Increases in sedimentary and algal turbidity are likely to result in altered underwater visual environments. We investigated the effect of these turbidities on the ability of two fishes, emerald shiner and walleye, to detect contrast underwater. Our findings suggest increases in suspended algae compromise vision more relative to sediment.

## Introduction

Visual ecology encompasses the physiological, evolutionary and environmental elements that enable visual cues to be detected by a receiver. Alteration of the visual environment is therefore expected to disrupt visually-mediated activities such as foraging, predator avoidance and reproduction (van der Sluijs *et al.*, 2011). In aquatic systems, anthropogenic particulate loading leading to increased turbidity is considered a primary threat to aquatic organisms, is known to alter the underwater visual environment, and has negative effects on aquatic biodiversity (Donohue and Molinos, 2009; Gray, 2016). Investigating the underlying mechanisms by which vision might be impaired due to elevated turbidity provides key information needed to predict the spatiotemporal population- and community-level shifts often associated with increased turbidity.

Turbidity causes light entering water to be scattered and absorbed by suspended particles, and therefore can lead to decreased light, shifts in the wavelength of light underwater, and blurred resolution of object borders being viewed (Lythgoe, 1979; Utne-Palm, 2002). Decreases in intensity and shifts in wavelength of light (i.e. the underwater environment becoming darker and differently colored, respectively) can significantly influence the ability of species that rely on vision to meet their ecological needs (Utne-Palm, 2002). Previous studies have shown that decreases in light availability often decrease an organism’s visual field and reaction distance (i.e. the maximum distance at which an object can be visually perceived; Miner and Stein, 1996; Utne-Palm, 2002; Richmond *et al.*, 2004; Radke and Gaupisch, 2005; Wellington *et al.*, 2010). Wellington *et al.* (2010) found that juvenile yellow perch (*Perca flavescens*) prey consumption was lower in phytoplanktonic (i.e. algal) turbidity than in sedimentary turbidity; however, consumption declined in all types of turbidity (e.g. algal and sedimentary). In another example, reduced visibility caused by increased turbidity resulted in a delay in the ontogenetic shift of Eurasian perch (*Perca fluviatilis*) from planktivory to piscivory (Radke and Gaupisch, 2005). Thus, we can expect both individual- and population-level impacts of turbidity on fishes reliant on visual cues for survival and reproduction.

Low levels of turbidity are natural in most waterbodies, and in some cases moderate to low turbidity results in higher performance of an individual via increases to contrast between the prey item and the background, especially when the prey are transparent plankton (Utne-Palm, 2002; Horppila *et al.*, 2004; Pangle *et al.*, 2012). Waterbodies that historically have had lower turbidity, however, are now experiencing elevated and more persistent turbidity events (Kane *et al.*, 2014; Gray, 2016). Anthropogenically-elevated inorganic (sedimentary) turbidity is typically the result of urban and agricultural surface water runoff (Mallin *et al.*, 2009), dredging, deforestation, erosion and mining (Wantzen and Mol, 2013; Gray, 2016). Many fine sediments remain suspended in the water column (i.e. sedimentary turbidity), and can also be re-suspended after major events, such as storms, thus raising the severity of turbidity. Increasing anthropogenic nutrient loading, on the other hand, causes drastic increases in the growth of suspended algae (i.e. organic, or algal turbidity), which in many lakes and rivers results in algal blooms (Michalak *et al.*, 2013). The production of toxins by some bloom-forming species of algae and cyanobacteria such as *Microcystis*, can result in algal blooms posing serious threats to the health of aquatic organisms and also to humans that utilize these waters (Ho and Michalak, 2015).

In general, we expect sedimentary and algal turbidity to differentially affect the underwater visual environment given the different light absorption and scattering properties of each type of particulate. Suspended sediments can vary drastically in size, shape and color depending on the local geological landscape, and typically resulting in greater scattering and loss of light at the ends of the visible spectrum (e.g. ultraviolet and red; Cronin *et al.*, 2014). Algal biomass, resulting in algal turbidity, tends to grow colonially near the surface causing gradation in the water column with higher densities near the surface (Klausmeier and Litchman, 2001) and because it contains photosynthetic pigmentation (i.e. chlorophyll), the light underwater will appear green (Cronin *et al.*, 2014). As the light attenuation properties of sediments are different than those of algae, the impacts of sedimentary and algal turbidity on visual ecology are not expected to be equal (Radke and Gaupisch, 2005; Wellington *et al.*, 2010). The consequences of these differences for the visual ecology of aquatic organisms are not well understood.

Changes to the visual environment, especially in historically clear waters, can act as a strong selective agent in visually-reliant fishes, resulting in both plastic and genetic modifications of fish visual systems. Evidence suggests that fish that rely on vision for reproduction and survival have visual systems tuned to the ambient light environment. For example, three spine stickleback (*Gasterosteus aculeatus*) from tannin-stained water tend to have red-shifted vision compared to populations found in clear lakes (Boughman, 2001). Under altered conditions, we might expect changes to visual sensitivity, even within very short-time frames. Rearing guppies (*Poecilia reticulata*) in turbid water, for example, led to a shift in wavelength-sensitive photopigments from mid-wavelength sensitive opsins (greens) to long wavelength-sensitive opsins (reds; Ehlman *et al.*, 2015). Shifting visual sensitivity in this way to match the background light environment may enhance contrast detection, motion detection abilities and increase foraging abilities under turbid conditions. However, not all species are expected to have such flexible visual sensitivity, thus elevated turbidity may significantly impair vision and visually-mediated behaviors in some species or contexts.

Turbidity can serve as a top-down control on community structure, reducing foraging efficiency of predatory trophic levels, or as a bottom-up control, significantly decreasing the depth of the photic zone, subsequently resulting in a decline in phytoplankton biomass (Aksnes and Giske, 1993). High levels of turbidity are often harmful to aquatic organisms, physically damaging respiratory structures such as gills (Sutherland and Meyer, 2007; Gray *et al.*, 2014), and negatively impacting foraging success and anti-predatory behaviors of visually oriented species (Miner and Stein, 1996; Abrahams and Kattenfeld, 1997; Ludsin *et al.*, 2001; Pangle *et al.*, 2012; Gray, 2016; Klobucar and Budy, 2016). In reservoir systems, high levels of turbidity have been found to reduce lake trout (*Salvelinus namayaush*) consumption of kokanee salmon (*Onchorynchus nerka*) to levels below the physiological demands of these fishes (Klobucar and Budy, 2016). Abrahams and Kattenfeld (1997) found an increase in risky behavior of fathead minnow (*Pimephales promelas*) in slightly turbid waters (mean ± SE = 11.01 ± 0.34 NTU nephelometric turbidity units [NTU]), as compared to clearer water (<1 NTU), resulting in reductions of the size selectivity seen by yellow perch predators in clear waters. In Eurasian perch, predation success is highly influenced by size and type of suspended particulates, as opposed to prey size (Radke and Gaupisch, 2005), likely resulting in alterations to trophic interactions in this highly visual predator.

Low levels of turbidity may be beneficial to some planktivorous fishes, as encounter rate with their planktonic prey will not change, while the likelihood of being predated upon decreases with increasing turbidity (Giske *et al.*, 1994; Abrahams and Kattenfeld, 1997). There is evidence that piscivorous fish should be disproportionately affected by turbidity, as their prey must be more visible at a larger distance than the small-bodied zooplankton that planktivorous fish predate upon (De Robertis *et al.*, 2003). For example, Shoup and Wahl (2009) found that largemouth bass (*Micropterus salmoides*) placed in high levels of turbidity (i.e. 40 NTU) select different prey items than bass in lower turbidity treatments (i.e. 0 and 5 NTU), with a significant decrease in foraging return at high turbidities. There is also some evidence that certain piscivorous fish species will increase their feeding activity in moderately turbid conditions. This is likely due to the increase in prey contrast with ambient background light (Boehlert and Morgan, 1985; Bristow *et al.*, 1996; Utne-Palm, 2002), as well as the decrease in likelihood of potential predation risk (Gregory and Northcote, 1993); however, this likely depends on the type and severity of the turbidity. The examples above, and the burgeoning literature on the effects of human-altered environments on predator-prey interactions across a number of systems (e.g. Miller *et al.*, 2017; Santonja *et al.*, 2017; Bastille-Rousseau *et al.*, 2018), demonstrate the difficulty in making predictions about how such dynamic relationships might be affected by environmental change. Thus, determining the responses of fishes from different trophic levels to environmental stressors will contribute to our ability to predict population- and community-level changes.

Lake Erie has been identified as impaired by multiple US National and Federal agencies, with the main threat being excessive loading of sediment and nutrients (Ohio Lake Erie Commission, 2016). Over the past century, changes in turbidity have altered species composition as well as species abundance in Lake Erie (Holm and Mandrak, 2002). This has in turn affected the dynamics of commercial and sport fisheries, including location and size of desired populations. Ludsin *et al.* (2001) found that while some Lake Erie fishes (e.g. black crappie, *Pomoxis nigromaculatus*, bluegill, *Lepomis macrochirus*) are tolerant of eutrophic events, such as algal blooms that increase algal turbidity, other species exhibit declines in abundance under these conditions (e.g. channel catfish, *Ictalurus punctatus*, white crappie, *Pomoxis annularis*). However, the impacts of turbidity on aquatic life tend to be species-specific (e.g. Bonner and Wilde, 2002; Radke and Gaupisch, 2005; Donohue and Molinos, 2009; Dugas and Franssen, 2012; Gray *et al.*, 2014) and do not always impact different life history stages in the same way (Utne-Palm, 2002). This highlights the importance of accounting for individual variation in responses (e.g. testing across multiple stressors) to more fully understand the species-level responses to elevated turbidity.

Emerald shiner (*Notropis atherinoides*) is one of the most abundant species in Lake Erie (Hartman and Margraf, 1992; McKenna *et al.*, 2008; Pothoven *et al.*, 2009), and considered a dominant forage fish (Pothoven *et al.*, 2009). Emerald shiner have been shown to highly influence the structure and composition of zooplankton communities (Hartman *et al.*, 1992; Pothoven *et al.*, 2009), as well as compose a large portion of the diet for many species of commercial and recreational fisheries of interest (e.g. walleye). Emerald shiner rely heavily on vision for foraging, schooling and predator avoidance behaviors (Bonner and Wilde, 2002) and based on congeneric vision studies are expected to have color vision (Bridges, 1964; Bowmaker, 1995). Bonner and Wilde (2002) found that stream populations of emerald shiner exhibit a decrease in prey consumption in sedimentary turbidity compared to clear water. The impact of turbidity on visual sensitivity thresholds has yet to be elucidated, and likely greatly influences the location and fitness of this species within Lake Erie.

Walleye (*Sander vitreus*) are a top predator in the Lake Erie ecosystem. Walleye are an economically important species in the area, as the recreational fishery in Lake Erie is the largest walleye fishery in the world, valued at $1.9 billion dollars annually (US Department of the Interior *et al.*, 2011). Walleye have adaptations in their visual sensory structures to accommodate foraging in low-light conditions. Specifically, they have a tissue layer, called a *tapetum lucidum*, that lays inside the retina and enhances scotopic, or low-light vision (Wahl, 1994). They are known to forage primarily in lower light levels, and foraging during daylight hours has been associated with low to moderate levels of turbidity (Ryder, 1977; Einfalt *et al.*, 2012). This fits with theoretical predictions that low to moderately turbid water might enhance the contrast of prey against the background, making it an easy target for predators (e.g. Utne-Palm, 2002; Pangle *et al.*, 2012). However, it is likely that too much light scattering in turbid water will pose a threat to the visual abilities of walleye at higher turbidity levels.

Our first objective was to test the visual sensitivity of these two ecologically and economically important Lake Erie fishes, the planktivorous forage fish, emerald shiner and a primary predator, the piscivorous walleye, under sedimentary and algal turbidity. We additionally aimed to compare the visual abilities of emerald shiner and walleye across turbidity types, given their distinct trophic positions. To determine visual sensitivity thresholds for each species under different types of turbidity, we used an optomotor response apparatus which utilizes the innate optikinetic response of an animal (i.e. following a moving object with the eye). This manifests as an optomotor response, which is the continual following of a moving object by an organism. The optomotor response is taxonomically widespread and has been observed in many species of fish, such as zebrafish (*Danio rerio*) and medaka (*Oryzias latipes*; Mueller and Neuhauss, 2010), guppies (*Poecilia reticulata*; Anstis *et al.*, 1998) and cichlids (*Pundamilia* spp.; Maan *et al.*, 2006), as well as other taxa including insects (McCann and MacGinitie, 1965; Collett, 1980; Honegger, 1981), mice (Prusky *et al.*, 2004; Abdeljalil *et al.*, 2005), and birds (Eckmeier and Bischof, 2008). This methodology allows the quantification of the ability of an animal to distinguish contrast between a moving black stimulus against a white background, as the test subject will follow the moving stimulus as long as it can be visually distinguished from the background. As turbidity is increased, we expect the contrast between the signal and the background to decrease to a point at which the organism can no longer distinguish the contrast, known as the visual detection threshold, and indicated by the cessation of following behavior by the animal (Robson, 1966). We tested the optomotor response of individuals under increasing sedimentary and algal turbidity, as well as under a treatment that combined sedimentary and algal particulates. These treatments mimic conditions potentially experienced in Lake Erie, for example during spring runoff and storms (sedimentary turbidity), late summer algal blooms (algal turbidity), and storms or dredging activities when a bloom is present (sediment plus algal turbidity). We predicted that sedimentary and algal turbidity would elicit different visual detection thresholds due to the different ways that each type of suspended particulate alters the underwater visual environment. Further, we predicted that the visual detection thresholds of emerald shiner would differ from those of walleye based on the different trophic positions they occupy, as well as the different foraging strategies on which they rely. Specifically, walleye should have relatively higher detection thresholds under low-light conditions (i.e. given the presence of the *tapetum lucidum* in the eye). While these species may also utilize different sensory modalities to forage and avoid predation (e.g. lateral line), here we solely focus on the alteration to visual processes caused by increasing turbidity.

## Methods

Emerald shiner were collected between June and August 2016 and 2017 using seine and cast net methods off the shore of South Bass Island in the western basin of Lake Erie. Fish care and experimental trials took place at The Ohio State University’s Franz Theodore Stone Laboratory located on South Bass Island, Ohio. Fish were held in 40 L aquaria with filtered lake water and maintained at a mean temperature of 23.3 ± 2.1°C on a natural light regime. Juvenile walleye were collected using otter trawls set at approximately 9.1 m depth from June to August 2017 in the waters surrounding South Bass and Middle Bass Islands in the western basin of Lake Erie. Walleye were held in a 1660-l cylindrical tank with a flow-through system and coarsely filtered Lake Erie water maintained at a mean temperature of 21.7 ± 1.3°C on a natural light regime. All fish were collected and held under Ohio Department of Natural Resources permit #18–82 and The Ohio State University IACUC protocol #2014A00000055.

Experimental sedimentary turbidity was created by mixing 20 g of sieved and dried Lake Erie benthic sediments (collected with an Eckman grab) with lake water (9000 mg/l). Algal turbidity solution consisted of emulsified spinach (450 g) sieved through a 1-mm mesh. Spinach was utilized in a similar study, citing the similarity in color, size and light scattering properties of emulsified spinach relative to common algal bloom species (Wellington *et al.*, 2010). The ‘sediment plus algal’ treatment, hereafter ‘combination turbidity’, was created using a mixture of 25% sediment solution to 75% algal solution, which, due to the relative concentration of each solution, resulted in a mixture whereby sediment and algal turbidity were contributing roughly equally to the resultant turbidity (measured in NTU). To show the relative change in light intensity and spectral composition created across treatments, we measured down-welling absolute irradiance approximately 15 cm below the surface of the water for each of the three treatments at 20 NTU. A portable Jaz spectrometer (OceanOpticsInc.) and a 600-μm fiber optic cable with a cosin corrector attached were used to measure the light (see Gray *et al.*, 2011). We then calculated the intensity and distribution of light relative to clear water and found the wavelength of maximum intensity (*λ*_max_) for each treatment.

**Figure 1:**
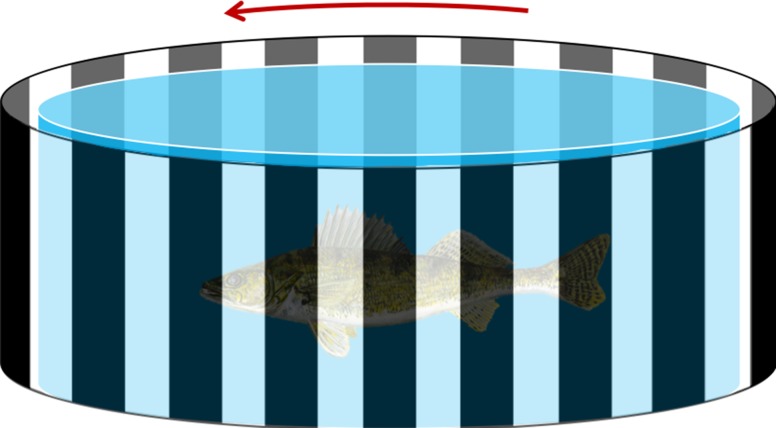
The optomotor apparatus consists of a cylindrical tank surrounded by a rotating screen which is situated approximately 2 cm from the edge of the tank. The screen has 3 cm repeating black stripe pattern for contrast. Fish will follow the black and white rotating stimulus until the turbidity level is such that the fish can no longer distinguish the contrast between the black stimulus and white background. Walleye were tested in a 40-cm diameter tank while Emerald Shiner were tested in an 18-cm diameter tank.

The optomotor apparatus consisted of a rotating screen with alternating 3-cm wide black and white stripes (Fig. 1). All trials were conducted between the hours of 10:00 am and 3:00 pm to utilize natural lighting and were recorded on digital video so that visual responses could be validated after trials were complete. Emerald shiner optomotor response trials were conducted in a cylindrical glass tank with a diameter of 18 cm and filled with 1500 ml of aerated lake water and surrounded by the optomotor screen at a distance of 2 cm from the outside of the tank. The optomotor screen was rotated at a speed of eight rotations per minute, as determined by pilot studies that confirmed the fish would follow the screen at this rate under clear conditions. Each individual emerald shiner (*n* = 17) underwent a total of four trials, two in sedimentary turbidity and two in algal turbidity. A subset of emerald shiner (*n* = 5) were also tested twice under a combination treatment. Walleye optomotor response trials were conducted in a 40-cm diameter cylindrical acrylic tank filled with 10 l of aerated lake water. The optomotor screen (2 cm away from the outside of the tank) was rotated at a speed of 12 rotations per minute, again based on a pilot study testing responsiveness of the fish to the rotating screen. All walleye (*n* = 6) were tested twice under each of the three conditions. Individuals of both species were randomly assigned a trial sequence using a random sequence generator so that the order of trials was random across the experiment. We also altered the direction of screen rotation between each of the trials within a treatment. Fish were given a 30-min acclimation period inside the test tank, the latter 15 min of which the screen was rotating. After the acclimation period, and only if the fish was following the screen, we added enough of the concentrated turbidity solution to increase turbidity by 4 NTU. This turbidity solution was added with a 3-ml pipette. We continued to add solution to increase turbidity by 4 NTU every 2 min (i.e. a turbidity step). Water samples were taken at each addition and turbidity measured using a LaMotte 2020e portable turbidimeter (accuracy ± 0.2 NTU; LaMotte Company, Chestertown, MD, USA). The turbidity step at which the fish ceased following the screen (i.e. swimming backwards, stopping completely or swimming haphazardly) was recorded as the response variable (i.e. visual detection threshold). Both species followed the rotating screen for greater than 2 h in clear water during pilot studies and did not tire, thus we do not consider the time of trials to have influenced the step at which fish stopped swimming. At the completion of all trials, fish were euthanized in an overdose of clove oil solution (1:10 eugenol:ethanol) according to standard operating practices. Weight (g), standard length (cm) and total length (cm) measurements were taken before preservation in 10% buffered formalin.

Generalized linear mixed models (GLMMs) were used to analyze trials (each species separately), with individual fish as a random factor to account for repeated use of each fish, as well as individual variation between fish; standard length and treatment were included as fixed effects. Post hoc Bonferroni–Holm correction of all-pair multiple comparisons was used to estimate pairwise comparisons within the statistical models. All statistical tests were conducted utilizing the statistical program R 3.4.2 (R Development Core Team, 2018), utilizing the linear mixed models in the R package lme4 (Bates *et al.*, 2015).

## Results

**Figure 2:**
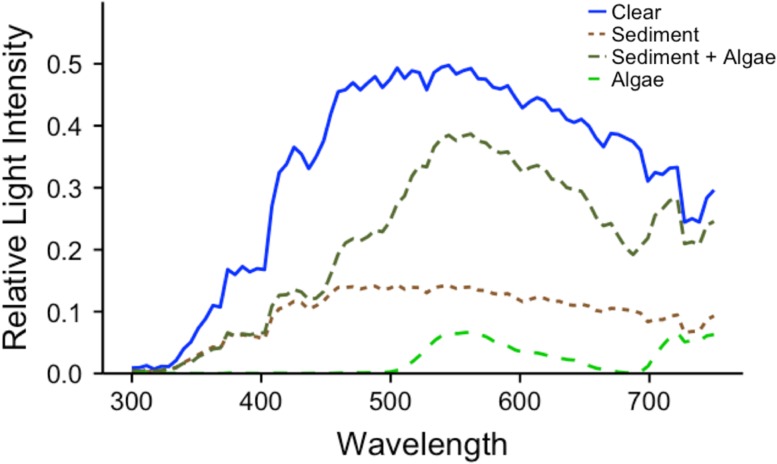
Relative (to ambient) light intensity spectra measured as down-welling irradiance 15 cm below the surface of the water in the optomotor tank in clear water (blue line), at 20 NTU of sedimentary turbidity (brown line), algal turbidity (green line) and a combination of algal and sedimentary turbidity (olive line). Natural daylight provided ambient light. Data was plotted using loess smoothing (span = 0.02).

Our measurements of spectral irradiance showed a decline in the amount of light and a shift in the dominant wavelength of light under different types of turbidity (Fig. 2). Sedimentary turbidity decreased the amount of light in the tank by approximately 35%, while algal turbidity reduced the amount of light by 42% and also shifted the dominant wavelength (*λ*_max_ = 506 nm) to a more green position (*λ*_max_ = 563 nm) indicative of high chlorophyll concentration. The combination treatment, having different concentrations of sediments and emulsified spinach, only decreased the amount of light slightly (~11%), but also green-shifted the light similar to the algae treatment.

**Figure 3:**
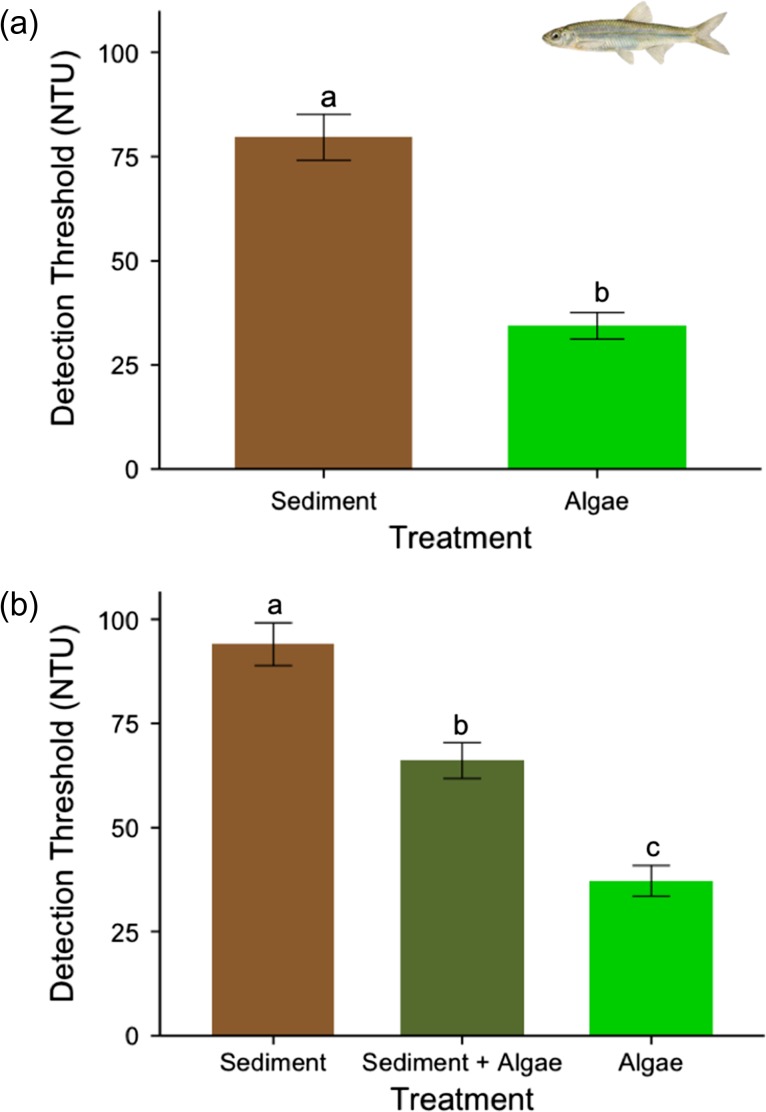
Mean (± standard error) detection thresholds for Emerald Shiner with individuals tested in (**a**) sedimentary turbidity (brown bar) and algal turbidity (green bar; *n* = 17, *P* < 0.001) and (**b**) all three treatments (subset *n* = 5) including a combination treatment (olive bar, *P* < 0.05). Each individual was tested twice under each treatments and the values for an individual within a treatment averaged. Post hoc pairwise comparison determined relationships between each treatment, as indicated by letters above bars.

Emerald shiner (standard length range: 4.1–6.2 cm) were determined to range from age 0 to 1, based on length–frequency analysis (Erzini, 1990). Model analysis showed that treatment significantly affected the visual detection threshold for emerald shiner (*n* = 17; Satterthwaite approx.; d.f. = 50, *F*= 65.791, *P* < 0.001). Visual detection thresholds for emerald shiner were significantly different between treatments (post hoc Bonferoni–Holm; d.f. = 50, *t* = −8.11, *P* < 0.001, SE = 5.57889, estimate = −45.25; Fig. 3a), with detection abilities diminished 43.2% in algal (mean_algal_ ± SE = 34.41 ± 3.19 NTU) compared to sedimentary (mean_sediment_ ± SE = 79.66 ± 5.51 NTU) turbidity. We also found variation in detection thresholds in the subset (*n* = 5; Fig. 3b; d.f. = 25, *F*= 65.766, *P* < 0.001) of emerald shiner that were tested under all three turbidity treatments (sedimentary [mean_sediment_ ± SE = 94.02 ± 5.14] NTU, algal [mean_algal_ ± SE = 37.21 ± 3.66 NTU] and combination [mean_combination_ ± SE = 66.10 ± 4.31 NTU] turbidity). Further analysis revealed that standard length was not on its own a significant indicator of detection threshold within each treatment (Satterthwaite approx.; d.f. = 5, *F* = 0.125, *P* = 0.737). Post hoc tests show that all three treatments were statistically distinct, with the highest detection threshold occurring in sedimentary turbidity (*z* = 5.557, *P* < 0.001), with combination turbidity the second greatest (*z* = 10.927, *P* < 0.001) and algal turbidity presenting the lowest sensitivity threshold (*z* = 5.370, *P* < 0.001). The relationship between standard length and visual detection threshold for the sediment treatment was found to be marginally significant (*t *= 2.039, *R*^2^ = 0.2171, *P* = 0.059), while length was not significant for any other treatment.

**Figure 4:**
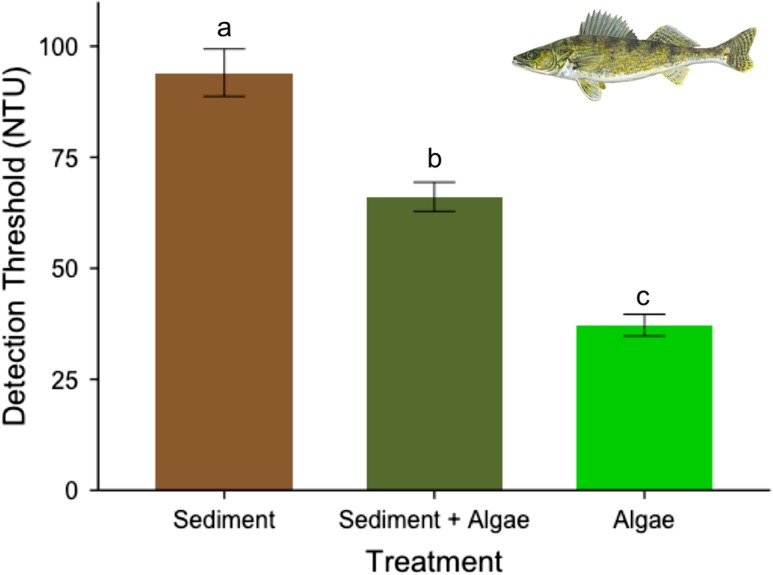
Mean (± standard error) visual detection thresholds for walleye (*n* = 6). Each individual was tested twice under each of three treatments and the values for an individual within a treatment averaged: sediment (brown bar), combination (dark green bar) and algal (light green bar) turbidity (*P* < 0.001). Post hoc pairwise comparisons revealed the relationships between each treatment, as indicated by letters above each bar.

Walleye ranged in size from 13.1 cm to 22.1 cm standard length and were all estimated to be less than 2-year-old juveniles based on size distribution for Lake Erie walleye (Crisovan, 2011). We found that treatment also significantly influenced visual detection thresholds in walleye (Satterthwaite approx., d.f. = 28, *F*= 53.037, *P* < 0.001), while standard length did not contribute significantly to the model (Satterthwaite approx., d.f. = 4, *F* = 3.107, *P* = 0.1527). Detection thresholds for walleye (*n* = 6; Fig. 4) were highest in the sedimentary treatment (mean_sediment_ ± SE = 99.98 ± 5.31 NTU), and decreased from the combination (mean_combination_ ± SE = 66.47 ± 3.27 NTU) to the algal (mean_algal_ ± SE = 40.35 ± 2.44 NTU) treatment, indicating that individual fish could detect the difference between the stimulus contrast at much higher levels of sedimentary compared to algal turbidity. Post hoc pairwise analysis revealed that these detection thresholds were statistically different, with the greatest being sedimentary turbidity (*z* = 6.432, *P* < 0.001), followed by combination turbidity (*z* = 14.686, *P* < 0.001), and with algal turbidity (*z* = 8.254, *P* < 0.001) having the lowest visual detection threshold. An analysis of the relationship between standard length and detection thresholds revealed a significant positive relationship between standard length and the combination turbidity treatment (*t*= 4.688, *R*^2^ = 0.8075, *P* = 0.009), however, there was no relationship between standard length and the detection threshold for either the sedimentary or algal treatments.

## Discussion

We found a drastic difference in the abilities of both emerald shiner and walleye to detect a moving stimulus dependent on the type of particulates used to create turbidity: detection thresholds were 43.2% and 40.4% lower, respectively, under algal turbidity compared to sedimentary turbidity in each species. These changes in detection threshold are likely due in part to the approximately 60 nm shift to longer wavelengths of light imposed by the algal compared to the sedimentary turbidity treatment and to the large decrease in the intensity of light, as measured at only 20 NTU (Fig. 2). Such differences in visual detection thresholds have been measured, for example, within and among individual cichlid fishes (Maan *et al.*, 2006) and between populations of guppies (Anstis *et al.*, 1998) using artificially altered colors and intensities of light; here, we demonstrate that individuals can detect and respond to ecologically relevant changes in the light environment as directly manipulated by increasing the concentration of different suspended particles in the water. We discuss the potential within- and between-species consequences of altered visual ecology imposed by elevated sedimentary and algal turbidity.

We tested the immediate response of individual fish to different types of turbidity and found markedly different detection thresholds among treatments. In emerald shiner, we found a 43.2% decrease in the ability to detect contrast within the optomotor apparatus when in algal compared to sedimentary turbidity, with a reduction from 80 to 34 NTU. Walleye had a similar change in visual detection pattern with the ability to detect contrast reduced from approximately 100 NTU in sedimentary to 40 NTU in algal turbidity, a 40% decrease in detection threshold. Body size showed some influence on detection thresholds for walleye in sedimentary turbidity but not the other treatments; the limited range of sizes tested here may not have been sufficient to detect a morphological relationship with turbidity. Regardless, our results suggest it is likely that the performance of both species at any visual task would be highly compromised by relatively low levels of suspended algae (~38 NTU). At this level, we found the cessation of optomotor response, meaning that even lower levels of algal turbidity could decrease the efficiency of visually-mediated behaviors such as foraging and predator avoidance. Juvenile yellow perch, for example, showed decreased foraging efficiency at only 20 NTU (Wellington *et al.*, 2010). Under mean levels of turbidity found in the western basin of Lake Erie (mean summer 2017 turbidity approx. 21 NTU; Great Lakes Environmental Research Laboratory, 2017) both species would likely be able to see. However, during storm events that lead to resuspension of sediments and turbidities exceeding 300 NTU (Great Lakes Environmental Research Laboratory, 2017) or algal blooms that can elevate turbidity to around or above 40 NTU (Great Lakes Environmental Research Laboratory, 2017) we can expect significant disruption to visually-mediated behaviors. Additionally, while lower levels of turbidity may not eliminate visual cues, it is likely that even moderate levels of increased turbidity are likely to impede visually-mediated behaviors such as foraging.

Another ecologically relevant form of turbidity in Lake Erie is a combination of sediment and algae, for example created by a late summer storm where sediments may be stirred up during an algal bloom event. We therefore tested a subsample of individuals in an additional combination treatment of both forms of turbidity. While we did detect variation across individual fish in their response to each treatment (i.e. individual as a random effect was included in the model), the pattern of decreased visual abilities from sedimentary to combination to algal turbidity is clear in both species; both emerald shiner and walleye in combination turbidity exhibited detection thresholds intermediate to those found in sedimentary and algal turbidity. In this case, we held the level of turbidity contributed by each type of particulate constant, rather than keeping the concentration of particles constant. This led to the combination treatment having higher light intensity compared to either the sedimentary or algal treatments, though the wavelength of maximum intensity shifted to match that of the algal treatment (at 20 NTU; Fig. 2). The higher light intensity therefore may have compensated for the wavelength shift, allowing for an intermediate visual response relative to the other treatments. Ultimately, this means that responses in nature are expected to vary depending on the relative concentration of different particulate types responsible for elevated turbidity and the inherent ways in which light is scattered and absorbed underwater. For example, algal blooms in Lake Erie are dominated by the cyanobacterium *Microcystis*, which form dense surface mats (Zohary and Madeira, 1990). These dense mats reduce the amount of light penetrating through the water column and change the color of ambient underwater light; however, when mixing of these mats occurs during heavy winds (i.e. algal cells become distributed throughout the water column), the algal particles could diffuse the light more uniformly, thus degrading the visual environment a slightly different way. Additionally, storm events that result in resuspension of sediments during an algal bloom may result in a combination of both sedimentary and algal turbidity occurring concurrently.

Predator-prey relationships are important for the functioning of ecosystems, and research to understand how species interactions are affected by human alteration of the environment is expanding (e.g. Miller *et al.*, 2017; Sanders and Gaston, 2018). With respect to turbidity elevated above natural conditions, predator-prey dynamics are expected to be influenced in different ways depending on the foraging strategy and size of organism (Abrahams and Kattenfeld, 1997). In this study, we tested each species in a size-specific optomotor apparatus with different speed and dimensions to accommodate size differences. This made a quantitative comparison of visual detection thresholds between species difficult; however, a qualitative assessment indicates some variation in the way that fish from different trophic levels respond to turbidity. Emerald shiner and walleye had relatively similar patterns of visual detection changes across turbidity type, indicating that trophic position may not necessarily be a driving force at the threshold of visual sensitivity (i.e. at the point where the fish can no longer detect contrast). While algal and combination turbidity affected the two species at a similar turbidity level, detection levels for walleye in sedimentary turbidity may be slightly higher, though further work is needed to evaluate this difference. The visual adaptations of walleye that give them an advantage under low-light conditions may contribute to their ability to detect contrast at higher sedimentary turbidity levels than emerald shiner. For example, the *tapetum lucidum* at the back of the walleye eye would be advantageous under the decreased light intensity resulting from sedimentary turbidity because it enhances light collection within the eye. However, shifts in wavelength of light, as expected with algal turbidity, may not be influenced by this adaptation. Until we better understand the species-specific photopic (color) vision sensitivities of walleye and emerald shiner it will be difficult to predict response to shifts in the wavelengths of water. The difference we detected between species with respect to sedimentary turbidity indicates that the consequences of elevated turbidity in general may have different impacts on fish populations and communities, though further work that examines individuals across a larger size range is warranted.

It is also possible that eye size differences, for example a larger walleye eye collecting more light than a smaller shiner eye, might also contribute to differences in responses. There were some indications that within-species, standard length (and hence eye size, assuming an allometric relationship) may play a role in visual detection abilities. This is possible given the relationship between eye size and visual acuity in teleosts (e.g. Wanzenböck *et al.*, 1996; Caves *et al.*, 2017, Corral-López *et al.*, 2017). For example, Dugas and Franssen (2012) found that populations of red shiner (*Cyprinella lutrensis*) from turbid waters have larger eyes than populations from clear water. Other examples show a negative relationship between eye size and turbidity (Huber and Rylander, 1992), suggesting concentration, duration and frequency of exposure may all be important factors in understanding visual responses to elevated turbidity. The direct functional role of eye size has rarely been experimentally tested in the context of elevated turbidity. It is likely that eye size highly influences visual abilities as teleost pupil size is fixed, and thus more light is capable of entering eyes with larger pupils, allowing greater visual processing under low-light conditions (Higgs and Fuiman, 1996). Fish length has been recently demonstrated to correlate with eye size, and visual morphology is known to relate to visual abilities (Caves *et al.*, 2017; Corral-López *et al.*, 2017). In our study, fish size did ultimately influence the best model for sensitivity threshold of walleye, even though on its own standard length was not significant. This was likely due to the relatively small sample size. In addition, it was only possible to test juvenile walleye with our current optomotor apparatus. More conclusions could be drawn about the effect of fish and eye size with a larger sample size that included adult post-recruitment walleye. An increased sample size would allow us to tease apart the effects of eye size on individual within-species variation in detection thresholds.

In general, there is a growing body of literature that seeks to understand behavioral responses to human-induced environmental change (e.g. Wong and Candolin, 2015; Sih *et al.*, 2011) and in particular how changes in species interactions might translate to shifts at higher levels of biological organization (Sih, 2013). Given that individuals of both species had different visual detection thresholds when tested under short-term exposure to different turbidity types in the optomotor apparatus, it seems fair to expect that increased turbidity fluctuations (e.g. from storm events enhanced by climate change; Whitehead *et al.*, 2009) and incidents of exposure (e.g. from more severe algal blooms; Michalak *et al.*, 2013) may have long-term consequences for populations of emerald shiner and walleye. Our study suggests that anthropogenically-induced algal turbidity may be more detrimental to the ability of fishes to function within that visual environment compared to sedimentary turbidity. Prolonged periods and increased density of algal turbidity are therefore likely to have severe consequences for populations of fish in Lake Erie that rely on vision for some or most of their life history and that remain in the western basin during intense algal blooms. Alternatively, visual foragers may begin to rely more heavily on alternative sensory modalities in order to find prey items (Sih *et al.*, 2011; van der Sluijs *et al.*, 2011). In addition, algal bloom events are associated with hypoxia (low dissolved oxygen) and higher water temperatures within the lake system, which can cause a variety of other negative consequences for aquatic species. For example, Arend *et al.* (2011) found a negative association between the quality of fish habitat and seasonal hypoxia occurrence in Lake Erie. Algal blooms may therefore pose significant challenges to fish in addition to altered visual environments leading to decreased foraging efficiency or a change in diet and consumption (Ludsin *et al.*, 2001; Pangle *et al.*, 2012; Klobucar and Budy, 2016), potentially resulting in decreased nutrition (e.g. if less preferable prey sources are more visible). Fish may also be likely to avoid areas in Lake Erie where turbidity is highest, leading to movement of populations out of the western basin during severe bloom years. In other systems, for example, juvenile coho salmon have been shown to behaviorally avoid high (>70 NTU) levels of turbidity (Bisson and Bilby, 1982). Avoidance responses were additionally found in six species of native New Zealand fishes at turbidity levels as low as 17 NTU (Boubée *et al.*, 1997). Further research is necessary to determine how patterns of decreased visual detection thresholds affect visual acuity and prey consumption, as well as the dynamic relationship between predator and prey. As both sedimentary and algal turbidity occur in the Lake Erie ecosystem, it is important for managers to understand the seasonal fluctuations of turbidity level and type, and be able to understand the dissimilar changes that will occur to fish dynamics in a multitude of situations.
